# Use of an advanced collagen matrix dressing on patients with complex chronic lower extremity ulcers: A case series

**DOI:** 10.1177/2050313X211013684

**Published:** 2021-05-13

**Authors:** Afsaneh Alavi, Jeannine Archer, Patricia Coutts

**Affiliations:** 1 Division of Dermatology, University of Toronto, Toronto, ON, Canada; 2 Mayo Clinic, Rochester, MN, USA; 3 York Dermatology Clinic and Research Center, Richmond Hill, ON, Canada

**Keywords:** Chronic wounds, venous ulcer, collagen

## Abstract

The objective of this case series was to assess the wound healing effectiveness of a collagen matrix wound dressing containing partially denatured collagen, carboxymethyl cellulose, alginate and ethylenediaminetetraacetic acid in chronic lower extremity ulcers. A total of nine patients with refractory lower extremity ulcers were treated with the collagen contact layer in addition to standard of care. Wound healing progress was measured at 2, 4 and 8 weeks. An average decrease in wound size of 73% was achieved across patients at week 8, with complete healing in two patients. The intervention was easy to use and well tolerated by patients. The results of this study, although preliminary, suggest that the advanced collagen matrix dressing represents an effective and safe treatment strategy for healing refractory chronic lower extremity ulcers of varying etiologies. Further investigation is needed to evaluate efficacy in a larger randomized clinical trial with focus on cost-effectiveness and impact on patient’s quality-of-life.

## Introduction

Chronic wounds are a clinically challenging and economically burdensome healthcare issue.^1,2^ Global rates of vascular disease and diabetes continue to rise and lower extremity wounds such as diabetic foot and venous insufficiency ulcers are affecting a growing number of the population worldwide.^3^ Lower extremity chronic wounds are challenging to heal with a high rate of recurrence. Even with compression therapy and appropriate care, venous leg ulcers (VLU) commonly persist for extended periods of years at a time.^4,5^ Many patients with lower extremity ulcers suffer from debilitating pain, recurrent infection, impaired work productivity and eventually poor quality of life.^6^

Under normal wound healing processes, proteases play an important role in the remodeling of extracellular matrix (ECM) necessary for timely progression through the phases of wound healing. However, elevated or prolonged expression of proteases such as matrix metalloproteinases (MMPs) can degrade newly formed tissue keeping the wound in a stalled state.^7^ To restore the wound healing process, clinicians often employ the use of bioactive dressings which interact with the wound environment to address imbalances among the increased MMPs and decreased tissue inhibitors of metalloproteinases (TIMPs). Protease imbalance augments degradation of the ECM, impairs cell migration, and reduces fibroblast proliferation and collagen synthesis, key processes essential to healing. Growth of new dermal tissue is a complex and dynamic process that can benefit from use of collagen-based dressings that interact with the wound bed to mediate elevated protease activity and stimulate the production and deposition of new tissue. As a main component of the ECM, collagen plays a critical role in wound healing.^8^ Formulated with other biomaterials aimed at addressing protease activity and macroscopic properties of the wound environment, such as moisture balance, it provides a valuable opportunity to advance healing. Collagen-based matrix dressings are indicated for a range of wound types, and review of the literature suggests an overall increase in healing rates for diabetic foot ulcers.^9^ There is evidence that outcomes for acute wounds such as burns are positively impacted as well.^10^ The aim of this case series is to study the clinical effectiveness of an advanced collagen matrix wound dressing that contains collagen, alginate, carboxymethyl cellulose (CMC) and ethylenediaminetetraacetic acid (EDTA), with or without silver (Ag) in advancing the healing of challenging lower extremity chronic wounds.

### Case report

This case series was conducted at York Dermatology Clinic and Research Centre, an outpatient community dermatology and wound clinic. The study was approved by an independent ethics review board. Prior to enrollment, all patients gave written consent to participate and were informed of their rights to discontinue their involvement in the study at any time.

A total of nine consecutive patients with chronic leg ulcers were included in this case series and treated with the collagen wound contact layer composed of partially denatured collagen, alginate, CMC and ethylenediaminetetraacetic acid (EDTA), with or without silver (Ag) (ColActive^®^ Plus & ColActive^®^ Plus Ag, Covalon Technologies Ltd., Mississauga, Ontario, Canada). In addition to the collagen matrix contact layer, patients received standard care consisting of wound cleansing, debridement, standard wound dressing as well as compression therapy. The decision to use the silver containing matrix was based on clinician judgment. If clinical assessment, according to NERDS,^11^ revealed critical colonization, wounds were treated with the dressing containing silver, otherwise patients received the collagen dressing without silver. Patients greater than 18 years of age with a chronic lower leg ulcer present for longer than 1 month, ankle brachial index^12^ of greater than 0.65, toe pressure greater than 55, and able to tolerate compression therapy met the inclusion criteria and were eligible to participate in the study, unless they met the exclusion criteria of HbA1C greater than 12%, presence of untreated wound infections and/or pregnant or breast feeding. The contact layer was cut to the size of the wound and placed directly onto the wound bed following necessary debridement procedures. Secondary foam dressings and a compression system (3M™ Coban™ 2 Lite Two-Layer Compression System) were used with the collagen contact layer as standard of care 2–3 times per week. Study visits were completed at weeks 2, 4 and 8 following enrollment and initial baseline measurements were taken at week 0. Wound size was recorded in centimeters squared (cm^2^), calculated from measurements of its greatest length and width. At assessments, patients were asked about pain level and their comfort during and between dressing changes.

Analysis was performed as intention-to-treat to include case 5, a patient who was lost to follow-up following week 4. Another patient, case 6, was discontinued from the study at week 4 when their wound had fully healed.

**Figure 1.**
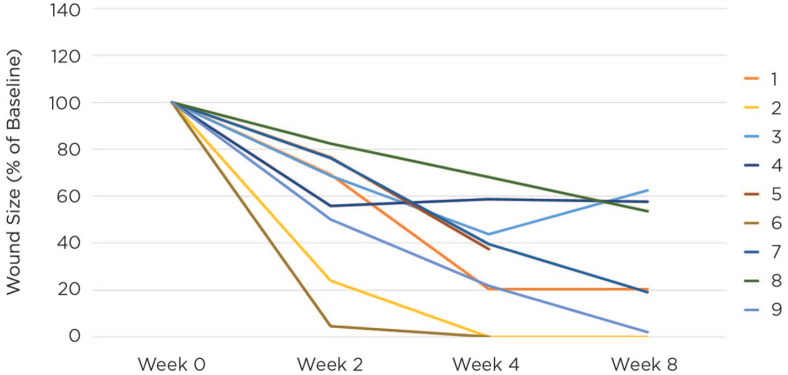
Wound healing over time.

**Figure 2.**
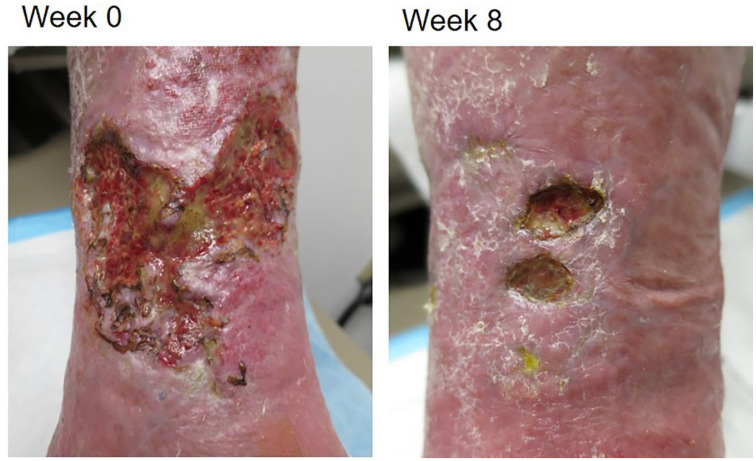
Pre (week 0) and post (week 8) images for case 2.

This case series included five men and four female patients with average age of 65.4 years. The average wound duration at enrollment was 24.9 months (Table 1). All patients tolerated the dressing well and no safety concerns were raised. After 4 weeks of treatment with the collagen matrix dressing in conjunction with standard of care, 68% wound closure was achieved across patients, progressing to 73% at the final visit or 8 weeks after the initial application. Two of the nine wounds healed fully over the course of the study, cases 3 and 7 were 100% healed at week 4 and case 1 by week 8 (Table 2). Figure 1 shows the progression of each case over the 8-week study period as wound size as a percentage of baseline. For cases 5 and 6, 8-week measurements are not available because the patients were lost to follow-up and achieved healing by week 4, respectively. All other wounds, despite their challenging nature, reduced significantly in size over the duration of the 8-week study (Figure 2; Table 2).

**Table 1.** Population characteristics and wound type.

Characteristics	Notes
Wound type	Venous leg ulcer
N	9
Female	4
Male	5
Mean age	65.4 (42-81)
Mean (range) wound size (cm^2^) at enrollment	11.31 (0.32-64.5)
Mean wound duration at enrollment (months)	24.9

**Table 2.** Case characteristics and outcomes.

Patient ID	Wound type	Product used	% Healed 4 weeks	% Healed 8 weeks	Diagnosis & co-morbidities
1	Venous leg ulcer	ColActive Plus Ag	80	80	Hypertension, environmental allergies, sleep apnea
2	Venous leg ulcer	ColActive Plus Ag	100	100	Eczema, food allergies
3	Venous leg ulcer	ColActive Plus Ag	55	37.5	Diabetes
4	Venous leg ulcer	ColActive Plus Ag	41	42	Hypertension, CAD
5	Venous leg ulcer & pyoderma gangrenosum	ColActive Plus	63	Lost to follow-up	Pyoderma gangrenosum
6	Venous leg ulcer	ColActive Plus Ag	100	N/A	Hypertension, arthritis, vasculitis
7	Arterial leg ulcer	ColActive Plus Ag	60	81	Hypothyroidism, peripheral arterial disease
8	Venous Leg Ulcer	ColActive Plus Ag	32	46	Hypothyroidism, hypertension, type 2 diabetes, hyperlipidemia, squamous cell carcinoma
9	Mixed venous/arterial leg ulcer	ColActive Plus Ag	78	94	hypertension, type 2 diabetes
Mean (range) % healed			68 (1–100)	73 (37.5–100	

## Discussion

The results of this case series show that treatment with the advanced collagen matrix dressing is associated with marked wound improvement over an 8-week period, suggesting that when used in combination with standard of care, this intervention may decrease the time to healing of chronic lower extremity ulcers. Collagen dressings interact with the wound bed to stimulate growth and provide a supportive scaffold for the deposition of new ECM.^8–10,13^ In addition to providing a supportive framework for growth, this advanced collagen matrix dressing utilizes multiple mechanisms to target elevated protease levels. Denatured collagen acts as a sacrificial substrate, sparing newly formed tissues from degradation by proteases, while EDTA specifically targets MMPs by binding zinc ions required for their activity. The CMC and alginate components of the dressing enable greater absorptive capacity than other collagen wound contact layers, to facilitate removal of protease-containing exudate away from the wound bed while maintaining moisture balance.^14^ The option to include silver for antimicrobial activity is useful for the management of critically colonized wounds and patients with a heightened risk of infection.

Chronic diabetic foot and venous leg ulcers are challenging to treat, often complicated by the management of underlying conditions and numerous comorbidities.^15^ The wounds included in this case series were of varying size, with complex local physiology and various systemic factors contributing to the challenging nature of the treatment. For example, two cases presented with medical histories of multiple wound etiologies including pyoderma gangrenosum and neuropathic ulcers. Nevertheless, with the use of the advanced collagen matrix, a bioactive approach, in conjunction with standard of care, we were able to progress healing and achieve favorable results for this patient population. Importantly, use of the dressing was simple to integrate into the clinic’s standard treatment regimens and dressing change schedules. Furthermore, it was easy to apply and well tolerated by patients.

Limitations to this study include a small sample size and lack of control cases. Further investigation is needed to evaluate efficacy in larger randomized clinical trial with focus on cost-effectiveness and impact of patient’s quality-of-life.

## Conclusions

The results achieved with use of the advanced collagen matrix contact layer which contains partially denatured collagen, alginate, CMC, and EDTA, with or without antimicrobial silver, in addition to standard of care, suggest that the product is safe and efficacious, offering an opportunity to accelerate healing in patients with refractory chronic lower extremity ulcers.
